# Repeatability and Reproducibility of Retinal Nerve Fiber Layer Parameters Measured by Scanning Laser Polarimetry with Enhanced Corneal Compensation in Normal and Glaucomatous Eyes

**DOI:** 10.1155/2015/729392

**Published:** 2015-06-22

**Authors:** Mirian Ara, Antonio Ferreras, Ana B. Pajarin, Pilar Calvo, Michele Figus, Paolo Frezzotti

**Affiliations:** ^1^Department of Ophthalmology, Miguel Servet University Hospital, Aragon Health Sciences Institute, 50009 Zaragoza, Spain; ^2^Department of Surgery, Gynecology and Obstetrics, University of Zaragoza, 50009 Zaragoza, Spain; ^3^Department of Family Medicine, Seminario Primary Health Care Center, 50009 Zaragoza, Spain; ^4^Department of Neurosciences, University of Pisa, 56100 Pisa, Italy; ^5^Department of Ophthalmology, University of Siena, 53100 Siena, Italy

## Abstract

*Objective*. To assess the intrasession repeatability and intersession reproducibility of peripapillary retinal nerve fiber layer (RNFL) thickness parameters measured by scanning laser polarimetry (SLP) with enhanced corneal compensation (ECC) in healthy and glaucomatous eyes. *Methods*. One randomly selected eye of 82 healthy individuals and 60 glaucoma subjects was evaluated. Three scans were acquired during the first visit to evaluate intravisit repeatability. A different operator obtained two additional scans within 2 months after the first session to determine intervisit reproducibility. The intraclass correlation coefficient (ICC), coefficient of variation (COV), and test-retest variability (TRT) were calculated for all SLP parameters in both groups. *Results*. ICCs ranged from 0.920 to 0.982 for intravisit measurements and from 0.910 to 0.978 for intervisit measurements. The temporal-superior-nasal-inferior-temporal (TSNIT) average was the highest (0.967 and 0.946) in normal eyes, while nerve fiber indicator (NFI; 0.982) and inferior average (0.978) yielded the best ICC in glaucomatous eyes for intravisit and intervisit measurements, respectively. All COVs were under 10% in both groups, except NFI. TSNIT average had the lowest COV (2.43%) in either type of measurement. Intervisit TRT ranged from 6.48 to 12.84. *Conclusions*. The reproducibility of peripapillary RNFL measurements obtained with SLP-ECC was excellent, indicating that SLP-ECC is sufficiently accurate for monitoring glaucoma progression.

## 1. Introduction

Progressive death of retinal ganglion cells and their axons in the retina leads to characteristic changes in the optic nerve head, which are the typical signs of glaucomatous optic neuropathy. These structural changes also result in functional visual field loss as measured by standard automated perimetry (SAP). Therefore, evaluating the retinal nerve fiber layer (RNFL) and monitoring its changes are key components in glaucoma management. Objective and quantitative assessment of the RNFL largely relies on digital imaging technologies, including scanning laser polarimetry (SLP).

SLP is an imaging technology used to measure the birefringence of the RNFL. Polarized light passing through a birefringent structure, such as the RNFL, experiences a phase shift (retardation) that is linearly related to the RNFL thickness [1]. The cornea and the lens also exhibit birefringent properties, which are neutralized in the SLP with variable corneal compensation (VCC) [2]. Although SLP-VCC usually compensates correctly the birefringence of the anterior pole, atypical birefringence pattern images may be observed in some cases [3]. Thus, the latest generation of SLP, SLP with enhanced corneal compensation (ECC), includes an enhancement module to improve the performance of SLP-VCC for detecting RNFL damage [4–7] and progressive RNFL changes [8].

SLP has a theoretical advantage in detecting changes because reduction of RNFL retardance resulting from disruption of the microtubules could be evident before the actual loss of nerve fibers due to injury of the optic nerve. The present study assessed the intravisit and intervisit reproducibility of peripapillary SLP-ECC parameters in healthy and glaucomatous eyes.

## 2. Methods

### 2.1. Subjects

The Institutional Review Board (Clinical Research Ethics Committee of Aragon, CEICA) approved the study design and all methods adhered to the principles of the Declaration of Helsinki. Informed consent was obtained from all participants. Healthy eyes were consecutively recruited from patients referred for refraction that underwent routine examination without abnormal ocular findings, hospital staff, and relatives of patients. The glaucoma group was recruited consecutively from an ongoing longitudinal follow-up study at the Miguel Servet University Hospital. This group included patients with primary open-angle glaucoma, pseudoexfoliative glaucoma, and pigmentary glaucoma. One hundred and forty-two white individuals were evaluated (82 healthy control subjects and 60 patients with glaucoma). When both eyes fulfilled the inclusion criteria, only one eye per subject was randomly included in the study.

Inclusion criteria were age between 18 and 80 years, refractive error not exceeding a 5-diopter sphere and a 3-diopter cylinder, best-corrected visual acuity of at least 20/25 (Snellen scale), and transparent ocular media. Participants with any history of cardiovascular, severe hematologic, or neuroophthalmologic disease, optic nerve abnormalities (e.g., tilted disc, drusen), or angle anomalies; any retinal disease (e.g., macular degeneration, diabetic retinopathy, and retinal detachment); or ocular surgery within 1 month of enrollment were excluded.

All subjects underwent a comprehensive ophthalmic examination, comprising a review of their medical and ophthalmologic history, determination of best-corrected visual acuity, slit lamp biomicroscopy, Goldmann applanation tonometry, central corneal ultrasonic pachymetry (OcuScan RxP; Alcon Laboratories Inc., Irvine, CA), fundus examination, and at least two reliable SAPs (24-2 Swedish Interactive Threshold Algorithm Standard examinations; Humphrey Field Analyzer, model 750i; Carl Zeiss Meditec, Dublin, CA). If fixation losses were higher than 20% or false-positive or false-negative rates were higher than 15%, the tests were repeated at least 3 days apart to avoid a fatigue effect. Abnormal SAP results were defined as typical glaucomatous defects with a pattern standard deviation significantly increased beyond the 5% level and/or a Glaucoma Hemifield Test result outside normal limits.

### 2.2. Classification into Groups

Healthy eyes were defined by an intraocular pressure of 21 mmHg or less and normal SAP. Glaucomatous eyes were defined as those with intraocular pressure readings of at least 21 mmHg and consistent abnormal visual field defects on SAP.

### 2.3. Scanning Laser Polarimetry with Enhanced Corneal Compensation Imaging

The same operator acquired the first three scans (15-minute intertest intervals) at the initial visit using the same SLP-ECC (GDx PRO, Carl Zeiss Meditec, software version 1.0) following a standard protocol to assess intrasession variability. A different operator obtained the fourth and fifth scans at two additional visits at least 4 weeks apart (±1 week) to assess intersession variability. All scans were acquired through undilated pupils with low ambient light. The participants kept their head still during each scan acquisition and looked at the internal fixation point to obtain the best alignment. A primary scan was captured before each calculation to compensate for the corneal birefringence.

The ECC mode introduced a predetermined large birefringence bias to shift the total retardation to a higher value to remove noise and minimize the effect of atypical patterns [9]. Following image acquisition, the birefringent bias was removed mathematically, point by point, from the final RNFL image. Calculations were performed on a ring of fixed-sized tissue centered on the optic disc automatically determined by the SLP-ECC software.

In this study, we excluded images that were obtained during eye movement. Only good quality images from SLP were accepted: centered and well-focused scans with a quality scan score higher than 6. SLP parameters included in the statistical analysis were nerve fiber indicator (NFI), temporal-superior-nasal-inferior-temporal (TSNIT) average, superior average, inferior average, and TSNIT standard deviation. Although some studies indicate that the NFI is the most sensitive parameter of SLP for glaucoma diagnosis [10, 11], its calculation method is based on various parameters and the result does not directly indicate RNFL thickness.

### 2.4. Statistical Analysis

All statistical analyses were calculated using IBM SPSS (version 20; IBM Corporation, Somers, NY) and MedCalc (version 12; MedCalc, Mariakerke, Belgium) statistical software. After checking for a normal distribution of variables, two-tailed Student's *t*-tests were used to calculate differences between normal subjects and patients with glaucoma.

The SLP measurement variability was assessed by the intraclass correlation coefficient (ICC), coefficient of variation (COV), and the test-retest variability (TRT). The ICC is a statistic that condenses the reproducibility of a parameter for a given group of subjects. A large ICC suggests small fluctuations among repeated measurements in the same individual. The ICC value can range from 0 to a maximum of 1 [12]. The COVs were calculated as the relevant standard deviation divided by the mean of the measurement values expressed as a percentage. TRT was defined as two times the standard deviation.

Repeatability was considered to be the variation in measurements acquired by the same operator under the same conditions at the same visit. Reproducibility was considered the ability of SLP to consistently obtain the same measurement performed by different operators at different visits. Thus, the intravisit analysis only included the three measurements obtained at the first visit, while the intervisit reproducibility included all five scans acquired during the study.

## 3. Results

### 3.1. Demographic Parameters

The present study comprised 142 subjects ranging in age from 38 to 76 years (mean 57.6): 60 eyes with stable open-angle glaucoma and 82 healthy eyes (control group). Other demographic and clinical characteristics of the sample are shown in Table 1.

### 3.2. Reproducibility of SLP-ECC Parameters

Table 2 shows the comparison of SLP parameters between the normal and glaucoma groups. Intravisit and intervisit ICCs were excellent for all RNFL parameters (Tables 3 and 4). TSNIT average had the highest values (0.967 for intravisit and 0.946 for intervisit measurements) in normal eyes, while NFI (0.982 for intravisit analysis) and inferior average (0.978 for intervisit analysis) had the best values in glaucomatous eyes. TSNIT standard deviation (0.928) and NFI (0.910) exhibited the lowest ICC values for the intra- and intervisit measurements, respectively, in the normal group. Superior average (0.920 for the intravisit and 0.917 for the intervisit analysis) produced the lowest ICCs in the glaucoma group. All COVs were under 10% for both the intravisit and intervisit measurements in both groups, except the NFI. TSNIT average had the lowest intravisit (2.43% in the normal group and 4.40% in the glaucoma group) and intervisit COVs (2.68% in the normal group and 4.71% in the glaucoma group). The TRT for NFI ranged from 6.48 to 6.55 in the normal group and from 10.61 to 12.84 in the glaucoma group. The intervisit TRT was 2.73 for the TSNIT average in the normal group and 3.93 in the glaucoma group. The TSNIT standard deviation had the lowest intervisit TRT (2.83) in the glaucoma group.

## 4. Discussion

The reproducibility of measurements obtained with any diagnostic test is key for diagnostic accuracy and for monitoring changes over time. Glaucomatous progression is typically slow, and, for that reason, it may be difficult to identify small changes during follow-up. The validity of a test for detecting this change depends on its ability to differentiate actual progression from the inherent variability among measurements. Quantifying measurement variability is, therefore, critical. Visual field assessment results are subject to long-term fluctuations, which limit the ability to detect glaucoma progression between two consecutive tests [13, 14]. On the other hand, while a series of fundus photographs can be used to evaluate changes in the optic disc, the subjective nature of this method and the requirement for experienced evaluators limit its accuracy for detecting progression as well as its general applicability [15, 16].

SLP assesses RNFL thickness around the optic nerve head. Because the technology is based on reflectivity, measurement is hampered by polarization of the ocular media, which can lead to measurement errors induced by non-RNFL birefringence. Improvements in this technology, including ECC, have led to more reproducible results and more accurate discrimination between healthy and glaucomatous eyes [17].

Although other investigators have evaluated the repeatability of RNFL measurements using SLP-ECC, the present study is unique in the fact that it demonstrates not only repeatability but also reproducibility of SLP-ECC parameters over time. Thus, our study design (measurements at 3 different visits) and 2 study groups (normal and glaucomatous eyes) provide new information regarding the reproducibility of SLP reported to date. We found that RNFL measurements acquired with SLP-ECC had low variability (high ICCs and low COVs) for healthy and glaucomatous eyes. These findings are consistent with those of Sehi et al. [7] who evaluated the repeatability of SLP-VCC and SLP-ECC. Mai et al. [18] evaluated the repeatability of RNFL measurements acquired with SLP-ECC in 16 normal subjects, 32 subjects with ocular hypertension, and 35 glaucoma patients and reported similar results but found that the measurement reproducibility in glaucomatous eyes was slightly worse than that in healthy eyes.

Although we also evaluated the reproducibility of the NFI, it does not seem to be the best parameter for detecting glaucoma progression. NFI is a machine-learning classifier based on a linear support vector machine, not a parameter to measure disease severity. We found that NFI showed the best ICC (0.982) in the intravisit session and extremely good intervisit session reproducibility (ICC = 0.975) in the glaucoma group, but in all cases with a worse COV. In fact, the Guided Progression Analysis software provided by the manufacturer does not rely on the NFI to compare measurements over time, but on the TSNIT average, superior average, and inferior average, as well as different maps and graphs.

Sánchez-García et al. [19] recently evaluated the repeatability of RNFL parameters measured with SLP-VCC in 75 normal eyes and reported good results. They compared the variability between SLP-VCC, Cirrus optical coherence tomography, and confocal scanning laser tomography. They observed less fluctuation between examinations with SLP-VCC, particularly in the superior RNFL. It should be noted, however, that their intravisit measurements were based on only two scans. Similar findings for intravisit variability were reported by Rao et al. [20], who assessed the repeatability of SLP-ECC in 140 eyes of 73 healthy subjects. Their COVs ranged between 1.7% (average TSNIT) and 11.4% (NFI).

Garas et al. [21] used the COV to assess the intravisit repeatability of RNFL thicknesses measured with RTVue-100 spectral-domain optical coherence tomography, SLP-VCC, and SLP-ECC in 37 eyes, including 14 normal or ocular hypertensive eyes and 23 eyes with moderate to severe glaucoma. COVs for the average thickness and the RNFL thickness in the four quadrants were less than 10% in eyes with moderate to severe glaucoma.

The present study has some limitations. First, only good quality images with a signal strength of at least 7 were included in the statistical analysis, which might have influenced the upper and lower limits of the variability of SLP-ECC parameters. Thus, our results can be applied to patients with moderate and good quality scans, while worse reproducibility results may be expected when diagnosing glaucoma progression based on a series that includes poor quality images. Further studies are needed to clarify the effect of low quality scans, such as those obtained in subjects with media opacities, which is common in daily clinical practice. Second, some glaucoma patients had previous experience with SLP testing, which might have contributed to the low variability observed in this group. This seems unlikely, because there is no evidence that SLP requires a training period due to a learning effect. Third, despite the fact that our sample comprised a wide range of glaucoma severities, our results may not extrapolate to all clinical situations [22].

In conclusion, intravisit and intervisit measurements of peripapillary RNFL obtained with SLP-ECC had excellent reproducibility. Clinicians must take into account the reproducibility of every SLP-ECC parameter to differentiate variability and true progression when monitoring patients with glaucoma.

## Figures and Tables

**Table 1:** Demographic and clinical characteristics of the sample.

	Control (*n* = 82)				Glaucoma (*n* = 60)				*p* ^*^
	Minimum	Maximum	Mean	SD	Minimum	Maximum	Mean	SD	
Age (y)	38	70	55.73	6.92	43	76	58.27	8.95	0.091
BCVA (Snellen)	20/25	1	0.92	0.09	20/25	1	0.90	0.10	0.263
IOP (mmHg)	13	20	17.95	2.06	23	45	27.69	5.87	<0.001
CCT (*μ*m)	476	619	563.53	36.58	470	600	533.49	28.68	<0.001
MD SAP (dB)	−1.48	1.75	−0.31	1.16	−29.74	−1.28	−7.04	7.04	<0.001
PSD SAP	0.94	1.86	1.42	0.21	2.05	14.33	5.63	3.60	<0.001
VFI	98	100	99.51	0.64	14	97	85.72	18.80	<0.001

^*^Student's *t*-test between the control and glaucoma groups.

SD: standard deviation; BCVA: best-corrected visual acuity; IOP: intraocular pressure; CCT: central corneal thickness, MD: mean deviation; SAP: standard automated perimetry; PSD: pattern standard deviation; VFI: Visual Field Index.

**Table 2:** SLP parameters for the five tests performed in the normal and glaucoma groups.

	Control (*n* = 82)				Glaucoma (*n* = 60)				*p* ^*^
	Min.	Max.	Mean	SD	Min.	Max.	Mean	SD	
*Intravisit measurements *									
First measurement									
NFI	2	48	19.26	8.52	2	98	48.25	30.97	<0.001
TSNIT average	37.4	65.4	51.18	4.76	25.8	58.3	41.91	8.54	<0.001
Superior average	46.6	77.8	62.29	6.50	3.1	70.2	46.85	13.56	<0.001
Inferior average	45.9	87.4	63.83	7.31	26.2	76.2	52.46	11.96	<0.001
TSNIT SD	15	34.1	24.48	3.76	8.9	29.3	19.30	5.79	<0.001
Second measurement									
NFI	3	58	19.04	9.48	2	98	49.35	31.17	<0.001
TSNIT average	40	69	51.04	4.88	17	58	41.85	8.95	<0.001
Superior average	46	84	62.26	7.20	15	72	47.78	12.70	<0.001
Inferior average	49	79	63.58	6.94	13	76	51.27	13.61	<0.001
TSNIT SD	15	33	24.49	3.69	6	31	19.28	6.32	<0.001
Third measurement									
NFI	3	44	18.87	8.48	5	98	46.90	30.03	<0.001
TSNIT average	43	61	51.18	4.26	24	58	42.57	8.58	<0.001
Superior average	50	79	62.19	6.14	23	74	49.25	12.38	<0.001
Inferior average	52	79	63.99	6.68	26	76	52.88	12.06	<0.001
TSNIT SD	17	32	24.68	3.46	8	32	19.65	5.74	<0.001
*Intervisit measurements *									
Fourth measurement									
NFI	2	42	15.94	9.47	6	98	47.59	32.09	<0.001
TSNIT average	43	63	52.58	5.01	25	56	41.95	8.82	<0.001
Superior average	50	80	64.73	8.07	23	69	48.66	13.13	<0.001
Inferior average	53	82	65.92	7.23	28	73	51.93	12.37	<0.001
TSNIT SD	17	36	25.65	4.11	9	29	19.55	6.21	<0.001
Fifth measurement									
NFI	2	64	18.19	11.84	3	98	49.98	31.23	<0.001
TSNIT average	41	61	51.54	5.01	23	59	40.89	8.90	<0.001
Superior average	49	80	62.47	7.38	20	75	47.36	13.83	<0.001
Inferior average	48	83	64.90	7.80	27	72	50.91	12.28	<0.001
TSNIT SD	13	36	24.77	4.42	7	30	18.86	6.28	<0.001

^*^Student's *t*-test between the control and glaucoma groups.

Min.: minimum; Max.: maximum; NFI: nerve fiber indicator; TSNIT: temporal-superior-nasal-inferior-temporal; SD: standard deviation.

**Table 3:** Intravisit repeatability and intervisit reproducibility of SLP-ECC parameters in the normal group (*n* = 82).

GDx parameters	Intravisit						Intervisit					
	ICC	ICC 95% CI		*p*	COV (%)	TRT SD	ICC	ICC 95% CI		*p*	COV (%)	TRT SD
		Upper limit	Lower limit					Upper limit	Lower limit			
NFI	0. 935	0.906	0.956	<0.001	21.82	6.55	0.910	0.855	0.947	<0.001	19.91	6.48
TSNIT average	0.967	0.949	0.979	<0.001	2.43	2.51	0.946	0.912	0.968	<0.001	2.68	2.73
Superior average	0.940	0.913	0.959	<0.001	3.86	4.84	0.938	0.900	0.963	<0.001	3.88	4.90
Inferior average	0.944	0.919	0.962	<0.001	3.69	4.76	0.934	0.894	0.961	<0.001	3.82	4.84
TSNIT SD	0.928	0.896	0.951	<0.001	6.20	3.03	0.912	0.857	0.948	<0.001	8.54	3.56

ICC: intraclass correlation coefficient; CI: confidence interval; COV: coefficient of variation; TRT SD: test-retest variability; NFI: nerve fiber indicator; TSNIT: temporal-superior-nasal-inferior-temporal; SD: standard deviation.

**Table 4:** Intravisit repeatability and intervisit reproducibility of SLP-ECC parameters in the glaucoma group (*n* = 60).

GDx parameters	Intravisit						Intervisit					
	ICC	ICC 95% CI		*p*	COV (%)	TRT SD	ICC	ICC 95% CI		*p*	COV (%)	TRT SD
		Upper limit	Lower limit					Upper limit	Lower limit			
NFI	0.982	0.972	0.989	<0.001	19.38	10.61	0.975	0.958	0.985	<0.001	15.82	12.84
TSNIT average	0.977	0.961	0.986	<0.001	4.40	3.53	0.977	0.961	0.986	<0.001	4.71	3.93
Superior average	0.920	0.878	0.950	<0.001	7.42	6.65	0.917	0.863	0.952	<0.001	7.88	6.86
Inferior average	0.960	0.939	0.975	<0.001	5.68	5.48	0.978	0.964	0.987	<0.001	5.36	5.13
TSNIT SD	0.951	0.924	0.969	<0.001	9.11	3.23	0.970	0.951	0.983	<0.001	8.30	2.83

ICC: intraclass correlation coefficient; CI: confidence interval; COV: coefficient of variation; TRT SD: test-retest variability; NFI: nerve fiber indicator; TSNIT: temporal-superior-nasal-inferior-temporal; SD: standard deviation.
